# Structure over color: Diagnostic information in H&E images resides primarily in grayscale

**DOI:** 10.1016/j.jpi.2026.100646

**Published:** 2026-02-05

**Authors:** Leslie Dalton, Ian O. Ellis, Emad A Rakha

**Affiliations:** a Department of Histopathology, South Austin Hospital, Emeritus, Austin, TX, USA; b School of Medicine, University of Nottingham, University Park, Nottingham NG7 2RD, United Kingdom; c Pathology Department, Hamad Medical Corporation, Doha, Qatar

**Keywords:** Digital pathology, Hematoxylin and eosin (H&E), Color manipulation, Grayscale structure, Image compression, Artificial intelligence, Histopathological diagnosis

## Abstract

Color is a defining feature of hematoxylin and eosin (H&E)-stained histological sections; however, the extent to which diagnostic interpretation depends on color rather than grayscale-defined structure remains uncertain. We utilized systematic digital color manipulations and deep learning (DL) to interrogate the diagnostic contribution of color in H&E-stained slide-based breast cancer grading. H&E images were transformed into the YCbCr color space, enabling independent manipulation of luminance (Y) and chrominance (Cb, Cr). Four image variants were generated: grayscale, color-only, color-blind-friendly (CBF), and extreme color compression (XCC). CBF images were produced by transferring red-green information (Cr) into the blue-yellow (Cb) channel, whereas color-only images were created by fixing luminance at a constant value. XCC involved differential compression of luminance and chrominance, with chrominance compressed at an extreme ratio (1:1000). Image fidelity was assessed using multi-scale structural similarity (MS-SSIM). DL models (ConvNeXt) for breast cancer grading were independently trained and tested using each image variant. Quantitative assessment confirmed that structural information is primarily localized within the luminance channel. Grayscale, CBF, and XCC images demonstrated minimal loss of image fidelity (MS-SSIM > 0.95), whereas color-only images showed markedly reduced fidelity (MS-SSIM ≈ 0.15). DL predictions were highly concordant across original, grayscale, CBF, and XCC images (Spearman ρ > 0.9 for all comparisons), with all achieving area under the curve values ≥0.85. Although performance was reduced for color-only images, it remained higher than anticipated. Notably, extreme compression of color channels did not adversely affect image quality or model performance. These findings provide evidence that diagnostic information in H&E images resides primarily in structural features encoded by grayscale. The results suggest that diagnostic AI models can operate effectively without color information, that the emphasis on color normalization may be overstated, and that color data can be subjected to extreme compression with limited impact on diagnostic integrity.

## Introduction

For much of the twentieth century, pathologists were trained using black-and-white (grayscale) images reproduced in textbooks and journals, and diagnostic expertise mainly developed independent of color fidelity.^1^^,^^2^ Generations of pathologists became adept at interpreting tissue architecture, cytological detail, and stromal relationships from grayscale representations and translating these features to color microscopy at the bench. Notably, seminal work such as the original Nottingham histological grading article relied heavily on grayscale photomicrographs, yet conveyed the defining diagnostic features with clarity and precision.^3^

Despite this historical reliance on structure, the role of color in histopathological diagnosis has remained an implicit assumption rather than a systematic evaluation. This is exemplified by the ongoing debate regarding the ability of color-blind pathologists to practice diagnostic histopathology.^4^^,^^5^ Concerns persist that color discrimination is essential for accurate interpretation of hematoxylin and eosin (H&E)-stained sections, which form the backbone of routine histopathological diagnosis.^6^ However, most individuals with color vision deficiency exhibit red–green variants,^7^^,^^8^ whereas H&E staining is dominated by blue (hematoxylin) and pink (eosin) hues. These observations raise the question of whether color is truly diagnostic or instead facilitates visual comfort and efficiency.

The transition to digital pathology has enabled rapid and precise manipulation of color, creating new opportunities to interrogate its diagnostic contribution. Digital platforms allow instantaneous conversion of H&E images to grayscale, color-blind-friendly (CBF) palettes, or color-altered representations that decouple chromatic information from luminance. This technological flexibility also permits systematic evaluation of whether artificial intelligence (AI) models, trained on histological images, require color to achieve robust diagnostic performance. Specifically, can diagnostic deep learning models utilizing H&E-stained slides operate effectively on grayscale images, or even on images in which color information is substantially altered or redistributed? In essence, can diagnostic AI of H&E images be “color-blind”?

Parallel to these questions is the extensive literature on color normalization in digital pathology, where variation in staining intensity and hue across labs is viewed as a major confounder for both human interpretation and AI model performance.^9^, ^10^, ^11^, ^12^, ^13^ In this context, the deliberate construction of CBF images represents a purposeful deviation from conventional normalization strategies. If a diagnostically robust interpretation is preserved under such color redistribution, the necessity and emphasis placed on color normalization may warrant re-evaluation.

Finally, this work intersects with a broader and potentially high-impact consideration: the relative importance of color in image compression. Compression is essential for large-scale digital pathology workflows but inevitably risks loss of information.^14^^,^^15^ If diagnostic content resides predominantly in grayscale-encoded structure rather than chrominance, then color channels could be compressed far more aggressively than luminance without compromising diagnostic integrity. Such an approach could have substantial implications for data storage, transmission, and computational efficiency in digital pathology.

Together, these considerations motivated a systematic investigation into the diagnostic role of color in H&E images, using AI as an objective and labor-efficient surrogate observer. Rather than re-evaluating established human competence with grayscale histology,^6^ we leveraged AI to perform large-scale, unbiased comparisons of color-altered and grayscale representations. Breast cancer grading was selected as a representative analytical endpoint due to its morphology-driven nature and well-established histological criteria. This task allowed for an integrated evaluation of human visual perception, digital image processing, and AI-based performance assessment.

## Methods

### Image preparation and color manipulations

**Fig. 1:**
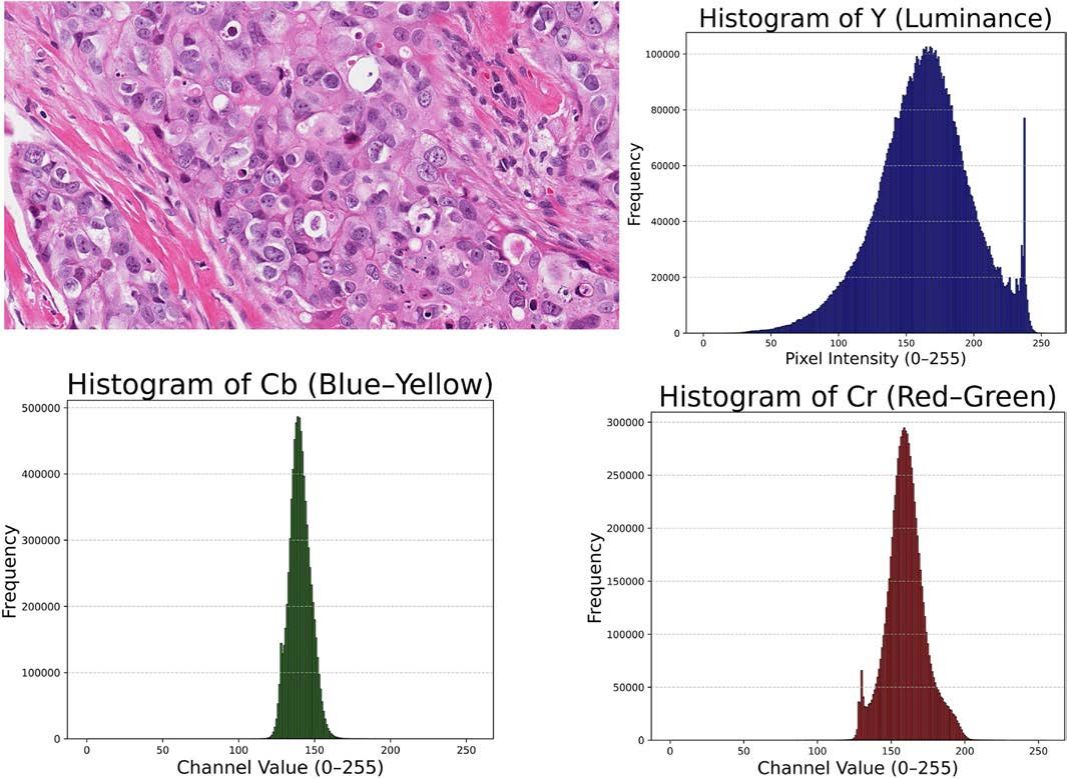
H&E image histograms illustrating dynamic range in the YCbCr color space. The luminance channel (Y) represents the broad dynamic range of grayscale intensities, with the right-side spike corresponding to transparent “glass” areas of the slide. In contrast, the chrominance channels (Cb and Cr) exhibit much narrower intensity bands, indicating they contain significantly less differential information compared to the structural grayscale component^.^

**Fig. 2:**
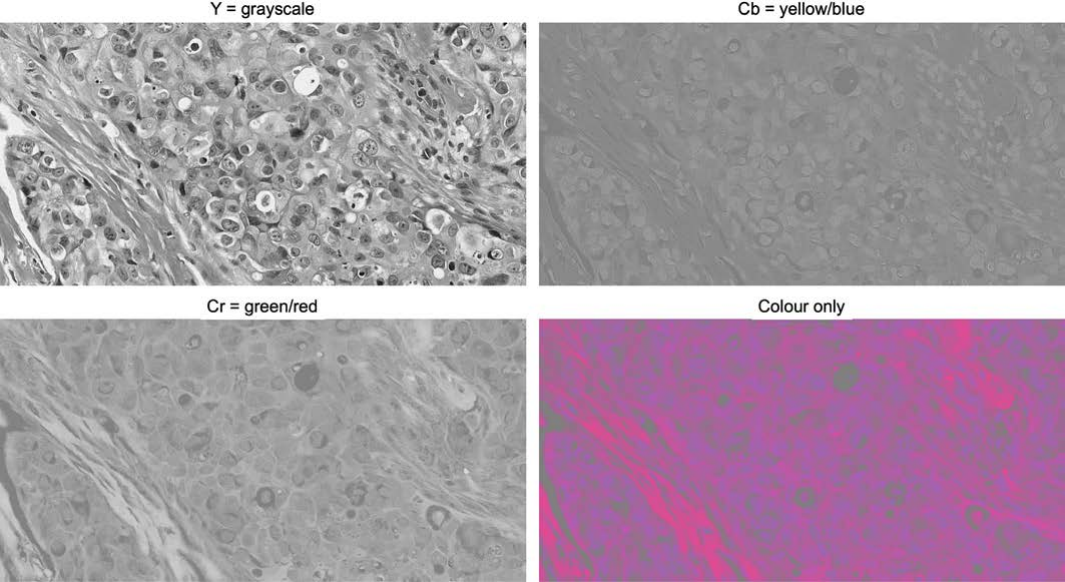
Decomposition of H&E images into discrete YCbCr components. The YCbCr color space separates an image into luminance (Y, grayscale) and two chrominance components: Cb (yellow–blue) and Cr (red–green). Cb and Cr are stored as 0–255 intensity values, yielding grayscale images (when luminance is held at a constant value, the remaining differential information is contained solely in the Cb and Cr channels). If Y is held constant, the only differential information is contained in Cb and Cr. Rejoining these fixed-luminance components results in the “color-only” image variant, which lacks traditional structural contrast. (For interpretation of the references to color in this figure legend, the reader is referred to the web version of this article.)

**Fig. 3:**
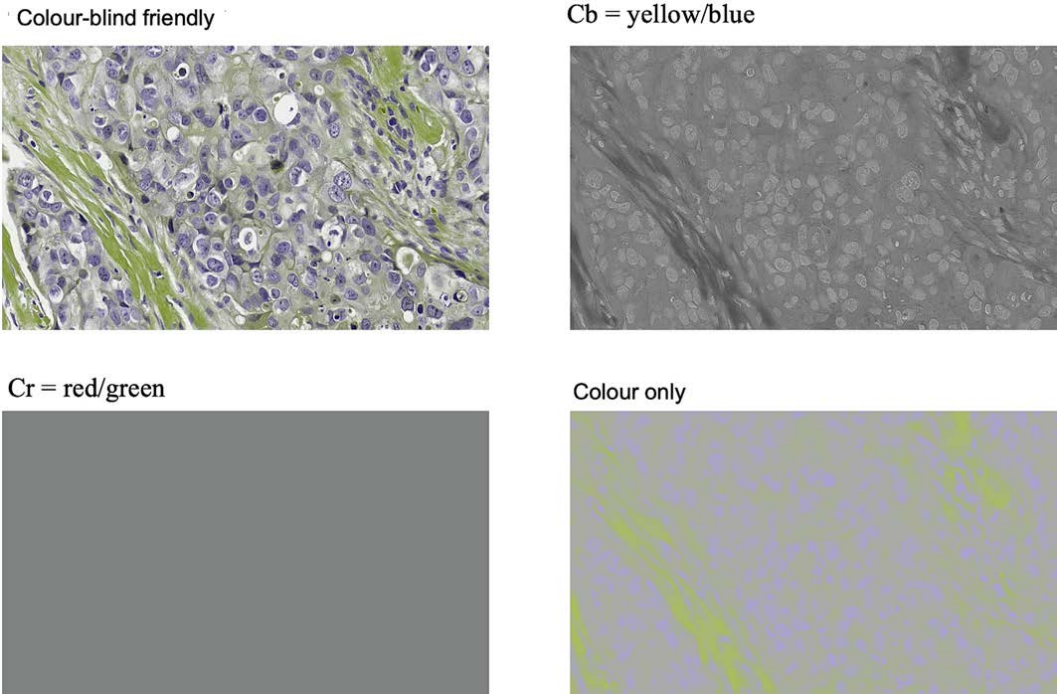
Implementation of the color-blind-friendly (CBF) variant. The left upper panel contains a color-blind-friendly image. To generate a palette perceptible to red-green color-deficient observers, differential information from the Cr channel was transferred to the Cb channel, with Cr subsequently set to a constant intensity. Differential red/green information was transferred to the Cb channel. Despite this chromatic redistribution, the resulting CBF and color-only images maintain identifiable features, such as nuclear highlights, which support both visual and computational interpretation. (For interpretation of the references to color in this figure legend, the reader is referred to the web version of this article.)

**Fig. 4:**
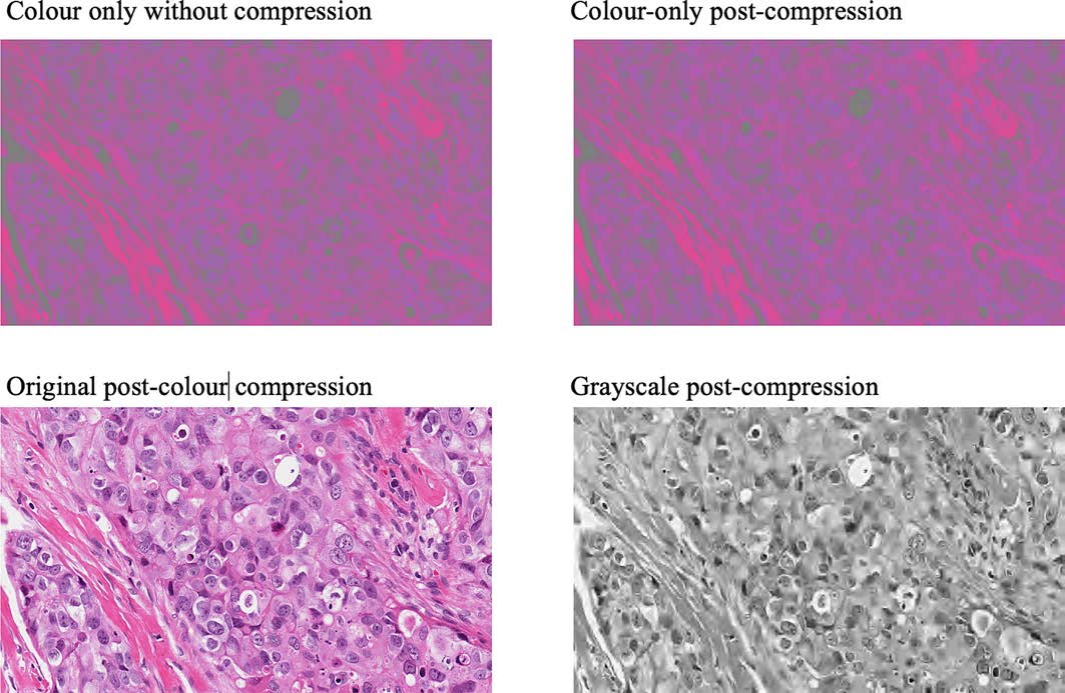
Impact of differential luminance and chrominance compression on image fidelity. This figure compares the consequences of aggressive data reduction within different channels of the YCbCr color space. *Upper left*: A baseline “color-only” image (fixed luminance) without compression. *Upper right*: The same color-only image following extreme chrominance compression (cratio = 1000); despite the massive reduction in data, there is no perceptible loss of chromatic information. *Lower left*: An eXtreme Color Compression (XCC) variant utilizing cratio = 1000 for chrominance but cratio = 1 (no compression) for grayscale structure. This illustrates that diagnostic morphology is preserved even when color data are selectively depleted. *Lower right*: A grayscale image (Y channel) subjected to the same cratio = 1000 compression. Significant degradation is visible as cytological details become “smudged,” confirming that grayscale information is structural and highly sensitive to compression-related loss^.^

**Fig. 5:**
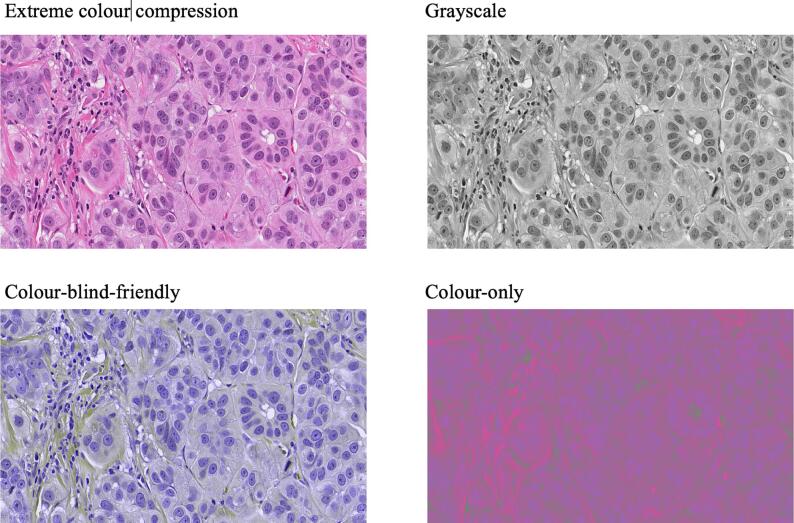
Comparative overview of the four experimental color alterations. This panel highlights the specific digital transformations used to decouple luminance from chrominance. *Upper left* (XCC): Despite having its color information (Cb, Cr) compressed at a ratio of 1000:1, the image appears virtually identical to the original H&E slide because the structural luminance component (Y) remains uncompressed. *Upperright* (Grayscale): The standard luminance-only representation (Y), which contains the dense architectural and cytological data required for diagnosis. *Lowerleft* (CBF): A color-blind-friendly representation where red–green differential information (Cr) was redistributed into the blue-yellow (Cb) channel, with the Cr channel set to a constant intensity. *Lowerright* (color-only): An inverse of grayscale where luminance is fixed at a constant value, leaving only the chromatic overlay; this results in a loss of nearly all structural contrast. (For interpretation of the references to color in this figure legend, the reader is referred to the web version of this article.)

All image transformations were generated by converting RGB tiles to the YCbCr color space, which separates luminance (Y) from chrominance (Cb, Cr).^14^^,^^16^^,^^17^ This enabled independent modification of grayscale structure and color information. Detailed algorithms and implementation parameters are provided in the Supplementary Methods. Four image variants were produced:

• Grayscale: Y component retained; Cb/Cr set to constants.
• CBF: Red–green information was redistributed into unused chrominance ranges, and Cr was flattened, producing a palette perceptible to red–green color-blind users.^18^^,^^19^
• Color-only: Y channel set to a constant value while Cb/Cr were preserved, removing all structural contrast.^13^^,^^20^
• Extreme color compression (XCC): Cb and Cr channels were compressed at very high ratios while Y was minimally compressed. This allowed selective depletion of color information while preserving morphology. Compression was performed using JPEG2000. Additional details, including compression ratios and quality-control steps, are provided in the Supplementary Methods. Representative examples of each transformation are shown in Fig. 1, Fig. 2, Fig. 3, Fig. 4, Fig. 5.

#### Image quality assessment

Structural preservation was quantified using the multi-scale structural similarity index (MS-SSIM), which approximates human perceptual assessment. MS-SSIM was calculated between each altered image and its corresponding original. Further explanation of metric selection and implementation is provided in the Supplementary Methods.

#### Dataset and deep learning models

Whole-slide images (WSIs) were sourced from The Cancer Genome Atlas (TCGA).^21^ Given the variable histological quality of TCGA WSIs, all images were reviewed by a pathologist (LD). Only slides considered to be of sufficient diagnostic quality, equivalent to that acceptable in routine clinical practice, were included. Training data comprised 192 breast cancer cases from 3 institutions; the external test set consisted of 271 cases from all remaining TCGA sites to ensure inter-institutional variability. In the training set, the median age was 58 years (IQR 19.8), whereas the test set had a median age of 60 years (IQR 21). The proportions of low-, intermediate-, and high-grade tumors in the training set were 18%, 39%, and 43%, respectively, compared with 20%, 45%, and 35% in the test set. Estrogen receptor–negative tumors accounted for 26% of the training set and 21% of the test set.

From these WSIs, pathologist-selected tiles representing high-grade, low-grade, and benign nuclei were extracted.^22^ Each image variant (original, grayscale, CBF, color-only, and XCC) was used to train separate convolutional neural network models (ConvNeXt-Tiny backbone pretrained on ImageNet).^23^^,^^24^ The task was to classify tiles according to the probability of containing high-grade nuclei. Slide-level scores were computed as the fraction of tiles classified as high-grade. Network architecture, augmentation, optimization parameters, and training procedures follow standard protocols and are detailed in the Supplementary Methods. Model performance was evaluated using receiver operating characteristic (ROC) analysis against a slide-level reference standard based on nuclear pleomorphism. Differences in areas under the curve (AUCs) were assessed by bootstrap resampling.

#### Brightness normalization

To avoid altering hue, brightness normalization was applied only to the luminance (Y) channel by scaling images to a common median grayscale value. Chrominance channels were left unchanged. Resulting MS-SSIM values remained >0.99, confirming minimal structural alteration (Supplementary Methods).

### Software

All image manipulation and model training were performed using Python and R.^25^ Key libraries included Pillow,^26^ glymur (JPEG2000),^17^^,^^27^ sewar (MS-SSIM),^28^, ^29^, ^30^ TensorFlow/Keras, and pROC.^31^ Full implementation details and reproducible code are provided in the Supplementary Methods.

Statistical analyses were performed in R.^25^ Spearman rank correlations were calculated using base R, with bootstrap confidence intervals generated using the boot package.^32^^,^^33^ ROC curves were constructed using the pROC package.^34^ The response variable was pleomorphism score 3 (high nuclear grade), and predictors were the model-derived probabilities of high-grade classification for each color alteration. Differences between AUCs, where present, were assessed using bootstrap resampling. Figures were generated using ggplot2.^35^

## Results

### Image fidelity, structural preservation, and normalization

Quantitative assessment confirmed that structural information is primarily carried by the luminance channel. Grayscale, CBF, and XCC images maintained high structural fidelity, with minimal perceptible degradation compared with the original full-color images. In contrast, color-only images initially resembled abstract patterns; however, on closer examination, some histological features remained recognizable, albeit with substantially reduced clarity compared with grayscale-based representations.

**Fig. 6:**
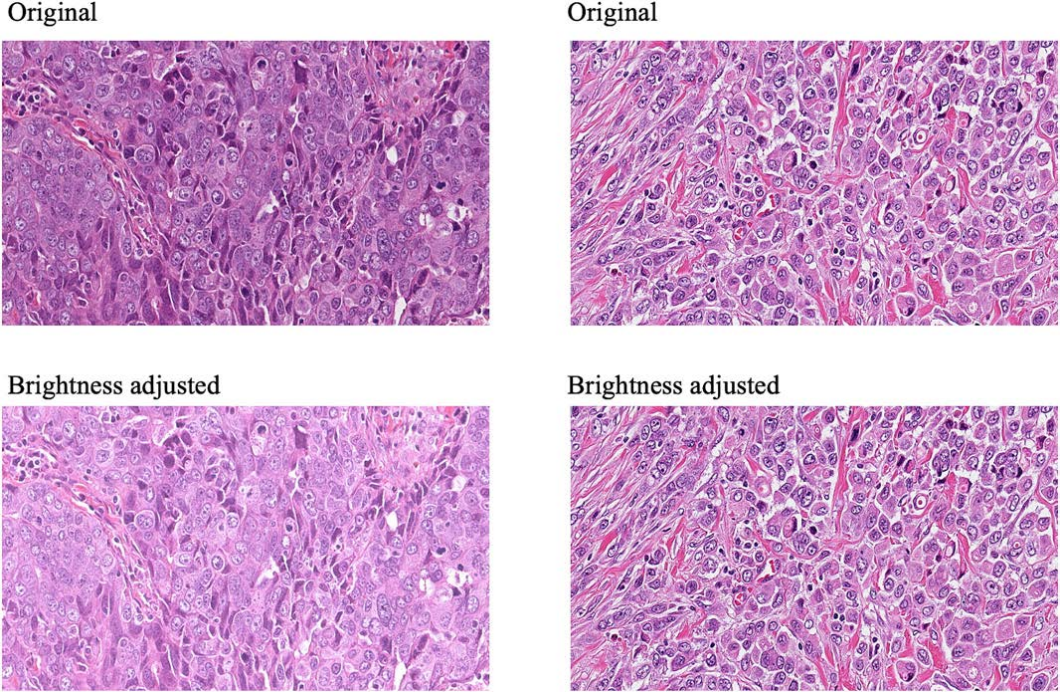
Luminance-only normalization for brightness standardization. This figure demonstrates a methodology for standardizing slide brightness without altering tissue hue or morphology. *Left panels*: An original H&E slide identified as excessively dark (Top) is harmonized to a reference median grayscale value (Bottom) by scaling only the Y channel. *Right panels*: Images with initial medians already close to the reference value show minimal change, demonstrating the stability of the scaling algorithm. *Structural preservation*: Because chrominance channels (Cb, Cr) are left untouched, the histochemical hue is preserved. As measured by the multi-scale structural similarity index (MS-SSIM), this normalization achieves values >0.99, indicating that luminance scaling corrects brightness disparities without compromising diagnostic detail.

Quantitative assessment using image quality metrics is summarized in Table 1. Relative to original full-color images, grayscale, XCC, and CBF images all demonstrated excellent structural fidelity, with median MS-SSIM values exceeding 0.95. An MS-SSIM value above 0.95 is generally regarded as indicating excellent image quality preservation.^29^ Notably, the use of luminance-only normalization (adjusting images to a uniform grayscale median while leaving chrominance unchanged) successfully corrected for brightness disparities in “over-dark” or “over-bright” slides without altering essential structural details (Fig. 6). Specifically, XCC images in which chrominance was compressed at a ratio of 1:1000, retained a median MS-SSIM of 0.97. Conversely, removing luminance (color-only images) resulted in a drastic loss of fidelity, with a median MS-SSIM of 0.148 (interquartile range 0.02–0.41), reflecting the loss of structural information when luminance was removed.

**Table 1:** Image quality metrics and AUC of deep learning models.

Color alteration	MS-SSIM (median [IQR])	AUC (95% CI)	*p*-value
Original full color	NA	0.85 [0.81–0.90]	NA
Grayscale	0.954 [0.88–0.98]	0.86 [0.81–0.90]	0.16
Color-blind-friendly (CBF)	0.951 [0.88–0.97]	0.88 [0.84–0.92]	0.02
Color-only	0.148 [0.03–0.42]	0.81 [0.76–0.86]	0.05
Extreme color compression	0.97 [0.95–0.98]	0.85 [0.80–0.90]	0.77

The area under the curve (AUC) corresponded to the ROC for the probability of a model to predict high-grade cancer. The *p*-value is whether the AUC of the color alteration was significantly different from the AUC of the original full color.

For XCC images, when the luminance channel was left uncompressed (Y cratio = 1.0), and chrominance channels were compressed at a Cb/Cr cratio of 1000, MS-SSIM remained high at 0.97 (0.95–0.98). Progressive compression of the luminance channel resulted in gradual degradation of image fidelity, with MS-SSIM falling below 0.95 at Y cratio values between 40 and 50. Importantly, no fixed MS-SSIM threshold is mandated, and user requirements and intended application can define acceptable compression levels.

### Model concordance and predictive performance

Deep learning model predictions were highly concordant across structural-preserving transformations. Spearman correlations between the original model and the grayscale, XCC, and CBF variants all exceeded 0.90. Specifically, the correlation between original and grayscale images was 0.95 (95% CI 0.93–0.96), between original and XCC images 0.94 (0.92–0.95), and between original and CBF images 0.91 (0.88–0.93). The correlation was lowest for color-only images (ρ = 0.81; 0.75–0.86), though it remained higher than anticipated.

**Fig. 7:**
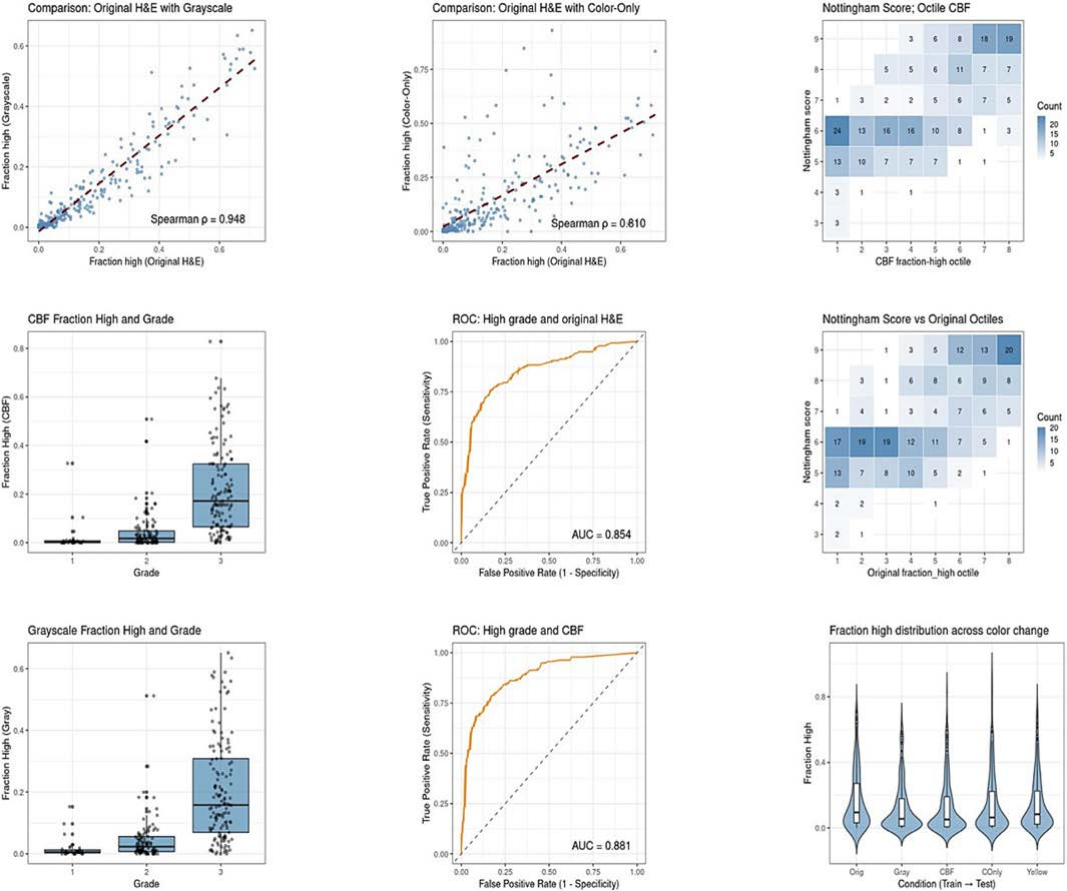
Comprehensive performance and concordance analysis across color-manipulated models. This multi-panel figure illustrates the comparative diagnostic utility and statistical agreement between the original H&E model and its digital variants. (A) *Correlation analysis*: Scatter plots demonstrate the linear relationship between probability predictions from the original H&E images versus grayscale and color-only variants. (B) *Predictive distribution*: Boxplots compare the distribution of probability predictions for grayscale versus color-blind-friendly (CBF) models across ternary pleomorphism grades. (C) *Diagnostic accuracy*: Receiver operating characteristic (ROC) curves evaluate high-grade pleomorphism detection, utilizing the “fraction high” (FracHigh) metric as the primary predictor. Results compare the performance of models trained on original versus CBF images. (D) *Spatial concordance*: Heatmaps provide a spatially resolved comparison of the original and CBF models. FracHigh values were discretized into octiles and correlated with the Nottingham histological grade. (E) *Global performance comparison*: A distribution plot summarizes the relative performance of all color-manipulated variants, highlighting the stability of model performance despite significant chromatic alterations.

**Fig. 8:**
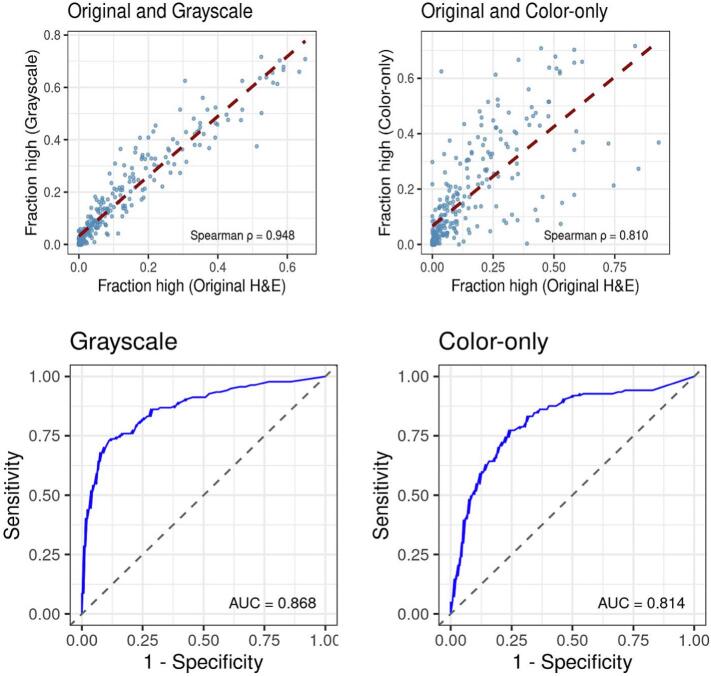
Quantitative comparison of grayscale and color-only diagnostic signals. This figure details the unexpected performance metrics observed when decoupling morphology from chromatic data. *Model performance*: Whereas the “color-only” model demonstrated surprisingly robust performance (AUC = 0.81) despite a significant loss of visible cytological detail, the grayscale-defined structural model yielded a significantly higher AUC (0.87). Concordance: The grayscale model showed a stronger linear correlation with the original full-color model than the color-only variant. *Statistical agreement*: Although not visually plotted in the scatter analysis, the Spearman correlation (ρ) between the grayscale and color-only outputs was 0.77, suggesting that whereas both channels capture diagnostic signal, the grayscale structural “blueprint” remains the primary driver of the deep learning model's predictive accuracy.

ROC analysis demonstrated robust model performance across the original, grayscale, CBF, and XCC images, with AUC values ≥0.85 (Fig. 7, Table 1). The original full-color model achieved an AUC of 0.85. The grayscale model demonstrated marginally higher performance and a stronger correlation with the original images than the color-only model (Fig. 8). Whereas the grayscale (AUC 0.87, *p* = 0.16) and XCC (AUC 0.85, *p* = 0.77) models were statistically indistinguishable from the original model, the CBF model showed a slight but statistically significant difference (AUC 0.88, *p* = 0.02). The color-only model yielded the lowest performance (AUC 0.81), reaching the threshold for statistical significance (*p* = 0.05) when compared to the full-color baseline.

A cross-tabulation showing the significance of delta (Δ) AUC across all permutations of color variations is presented in Supplementary Table 1. With the exception of a single comparison, all ΔAUC values were non-significant or borderline. The AUC for CBF images was significantly higher than for the color-only images (*p* = 0.0008). The basis for this difference is not apparent on subjective review and warrants further investigation.

### Practical implications: Color compression and luminance preservation

**Fig. 9:**
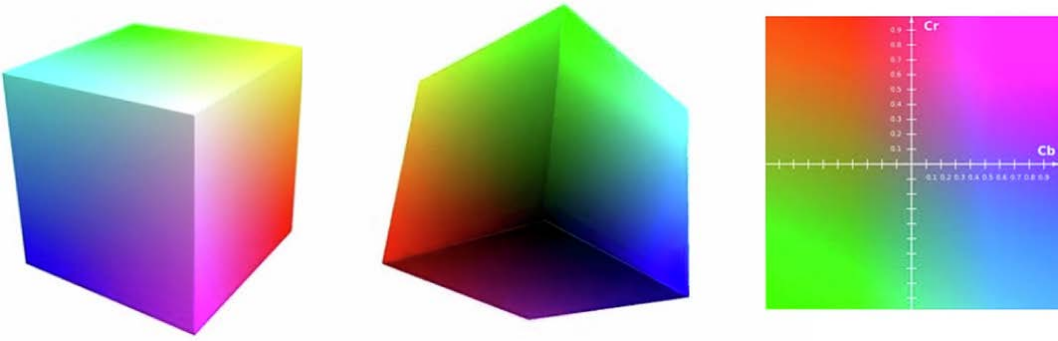
Separation of structure and color in the YCbCr color space. YCbCr is a perceptually motivated transformation of the RGB color space that isolates image data into a single luminance (Y) channel and two orthogonal chrominance (Cb, Cr) channels. In this model, the diagonal of the RGB cube defines the grayscale luminance axis, which encodes the essential tissue architecture and cytological detail, including nuclear morphology and spatial organization. The chrominance planes, analogous to color selection maps, primarily reflect H&E stain hue but contribute minimal structural contrast. Because diagnostic morphology is predominantly carried by the luminance channel, histological features remain visually and clinically intact even when color information is altered or substantially compressed.

For the practicing pathologist, the mechanism behind this efficiency is illustrated in Fig. 9. Digital histology images can be decomposed into the YCbCr color space, which isolates a luminance (Y) channel from two chrominance (Cb, Cr) channels. Critically, essential diagnostic features, including tissue architecture, nuclear size, pleomorphism, and spatial organization, are predominantly encoded within the luminance (grayscale) component. Chrominance primarily reflects H&E stain hue, which contributes significantly less to structural contrast. Consequently, diagnostic integrity is preserved even when color information is heavily compressed, suggesting that current digital storage and transmission protocols could be significantly optimized without compromising clinical safety.

**Fig. 10:**
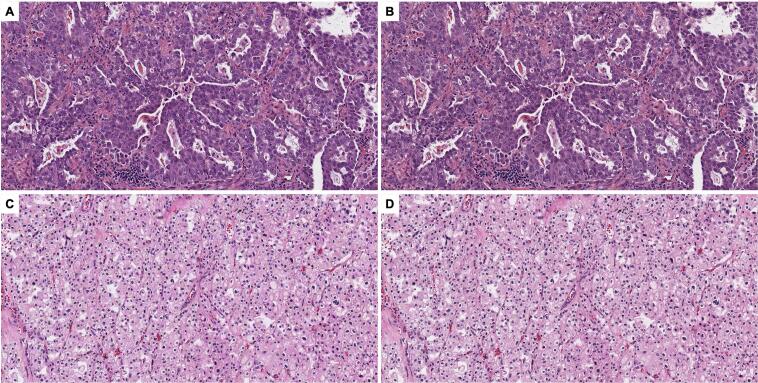
Extreme color compression (XCC) substantially reduces storage requirements while preserving image fidelity. The upper two images show lung adenocarcinoma; the lower two images show chromophobe renal cell carcinoma. Before compression, 216 high-resolution image patches (6000 × 6000 pixels) required 12.9 GB of disk storage (A and C). After application of XCC, the total storage was reduced to 404 MB, with the chrominance channels (Cb and Cr) contributing only 7.7 MB each (B and D). Overall, color information accounted for approximately 0.12% of the original storage footprint. Despite this degree of compression, image similarity remained high (median MS-SSIM = 0.965; interquartile range, 0.943–0.984), and visual assessment demonstrated only minimal perceptible degradation. Representative images are from lung adenocarcinoma.

XCC, which preferentially targets chrominance rather than luminance, substantially reduces data size while preserving image quality. The application of XCC achieved an overall file-size reduction of approximately 97%, while maintaining high visual fidelity, with a median MS-SSIM of 0.965. No meaningful loss of diagnostic morphology was observed on visual inspection, indicating that aggressive compression of color information had minimal impact on structural detail (Fig. 10).

## Discussion

Pathologists have long recognized that diagnostic information resides primarily in grayscale-defined structure, yet objective demonstration of this principle has been limited. It is not uncommon for intuitive insights to precede formal proof. A classic example is the four-color theorem, proposed by Guthrie in 1852^36^ to describe map coloring, but not formally proven until more than a century later, with the aid of computational methods.^37^^,^^38^

In this study, we explored multiple complementary ways of visualizing and manipulating color in H&E-stained images of breast cancer, some familiar and others less commonly considered. The first observation arose from a simple histogram analysis of the YCbCr components, which showed that the chrominance channels occupy a much narrower intensity range than the luminance channel. In contrast to grayscale, which spans the full dynamic range, the red–green and blue–yellow components contain relatively little differential information, suggesting that color contributes modestly to the discriminative signal. This finding is consistent with the biochemical basis of H&E staining. Unlike the broad chromatic diversity present in natural scenes, H&E images are derived from two dyes with highly specific binding properties.^39^ Hematoxylin, a basic (cationic) dye, binds primarily to negatively charged nucleic acids within nuclei, whereas eosin, an acidic (anionic) dye, binds to positively charged cytoplasmic and stromal proteins. These constrained chemical interactions inherently limit the range of colors produced, reinforcing the observation that color variation in H&E images is biologically and chemically restricted.

A second approach involved generating color-only images, conceptually representing the inverse of grayscale. At first glance, these images resemble abstract or impressionistic artwork; however, closer inspection reveals that some histological information is retained. Nevertheless, the loss of luminance markedly reduces clarity, supporting the interpretation that color alone functions largely as an overlay on grayscale-defined structure rather than as an independent carrier of diagnostic content.

We also examined CBF images. Although it is unlikely that this specific CBF implementation will become a routine diagnostic tool for pathologists with color vision deficiency, it served as a useful perturbation of chromatic information. That CBF images remained interpretable, both visually and computationally, argues against a strict dependence on conventional color representations and raises questions regarding the necessity of extensive color normalization in digital pathology workflows.

The investigation of XCC yielded particularly practical insights. Compression is a critical consideration in digital pathology, given the scale of WSI. Our results demonstrate that color channels can be compressed by orders of magnitude more than grayscale without perceptible loss of diagnostic morphology. In contrast, aggressive compression of the luminance channel rapidly degraded image quality, with cytological detail becoming unacceptable. These findings indicate that optimization of image compression should focus primarily on grayscale content, whereas color can be compressed far more aggressively with minimal consequence.

In the YCbCr color space, approximately two-thirds of the initial data allocation is devoted to chrominance. We showed that this chrominance component can be reduced to well below 1% of the original image size without compromising image quality. In this sense, color is computationally “cheap,” whereas grayscale structure is information-dense and must be preserved.

AI was not employed here to develop a clinical decision-support tool, but rather as an objective, quantitative probe of H&E image content. If color were essential for diagnosis, AI performance would be expected to deteriorate substantially when color information was altered or removed. Instead, model performance on grayscale, CBF, XCC, and original full-color images was statistically indistinguishable, demonstrating that AI models can operate effectively without color information. This provides objective support for the long-held intuition among pathologists that morphology, rather than color, underpins diagnostic interpretation.

Color-only images performed better than anticipated, suggesting that AI can exploit subtle chromatic patterns that are not readily perceptible to human observers. However, combining color-only and grayscale models did not improve performance. As previously described,^40^ ensemble methods require diversity and low correlation among base learners to yield benefit. The strong correlation between grayscale and color-only predictions explains the absence of additive value, further reinforcing the dominance of structural information.

Additional conceptual support for the primacy of structure comes from emerging work demonstrating that AI can extract diagnostically relevant information from unstained or minimally processed tissue sections. Using approaches such as autofluorescence imaging, quantitative phase microscopy, or label-free brightfield imaging, deep learning models have inferred tissue architecture, cellular features, and even molecular correlates without histochemical staining.^41^, ^42^, ^43^ Whereas such techniques are not intended to replace routine H&E assessment, they provide compelling proof-of-principle that meaningful histological information exists independent of color.

### Clinical relevance and limitations

High-grade nuclear feature detection was deliberately selected as a clinically meaningful surrogate task. Nuclear grading is central to routine breast cancer diagnosis and directly informs histological grading, prognostic stratification, and treatment decisions.^22^^,^^44^ It relies primarily on nuclear size, shape, chromatin texture, nucleolar prominence, and mitotic activity, features that are predominantly structural and contrast-based rather than color-dependent.^45^ As such, it represents one of the most diagnostically critical yet relatively color-independent assessments in histopathology, making it well-suited to interrogating the relative contributions of luminance and chromatic information in H&E-stained images. The preservation of AI model performance across grayscale, CBF, and extremely color-compressed conditions therefore has direct relevance to routine diagnostic practice and is consistent with prior human-observer studies demonstrating robust interpretation of grayscale histology. Practical implications for pathology practice, AI development, and digital pathology systems are summarized in Table 2.

**Table 2:** Practical recommendations for pathology, AI development, and digital pathology systems.

Stakeholder	Practical recommendations	Rationale/Expected benefit
Pathologists	• Prioritize structural (luminance) information when evaluating H&E images, recognizing that diagnostic features are largely preserved even when color is modified. • When developing QC guidelines, focus on brightness, contrast, and resolution before chromatic fidelity. • For teaching and QA, consider including grayscale or color-modified examples to reinforce structure-based interpretation. • Avoid over-reliance on color-based cues for tumor grading or nuclear assessment, as color variation between institutions is common.	• Supports robust interpretation across labs with variable staining. • Reinforces that morphological detail, not color, carries primary diagnostic value. • Helps reduce cognitive bias introduced by stain variability.
AI developers	• Design and train models with emphasis on luminance-based features, which are the dominant source of diagnostic information. • Incorporate color-agnostic data augmentation (e.g., grayscale conversion, channel perturbation) to improve model robustness. • Evaluate models under extreme color transformations to ensure generalizability across labs and scanners. • Document model sensitivity to color versus structure to ease regulatory review and clinical adoption.	• Enhances cross-site generalization and reduces model failure due to staining variability. • Encourages resilient models that perform consistently across color domains. • Facilitates regulatory approval by demonstrating robustness to pre-analytic variation.
Digital pathology vendors	• Optimize compression pipelines by applying higher compression ratios to chrominance (color) channels than to luminance channels. • Consider implementing selective color-channel compression in WSI formats (e.g., JP2K custom profiles). • Provide brightness and luminance-normalization tools rather than color-heavy adjustments in viewing software. • Transparently label color compression levels to support compliance with diagnostic imaging regulations.	• Reduces storage and bandwidth needs with negligible loss of diagnostic content. • Supports efficient telepathology and cloud workflows. • Aligns WSI compression with the biological importance of image components.

The better-than-expected performance of color-only images prompted consideration of the minimum visual information required by DL models to achieve better-than-random classification. Although color-only inputs lack luminance-encoded structure, they retain chromatic variation that may reflect stain uptake, chromatin density, or other biological or technical features. These residual cues were detectable both quantitatively (MS-SSIM) and on subjective review, where initially abstract color fields revealed subtle patterns such as nuclear clustering and cytoplasmic-to-nuclear variation. Whereas unfamiliar to a pathologist at first glance, these patterns become interpretable with repeated exposure, suggesting that chromatic information alone may carry an exploitable signal. These findings imply that the relative importance of color versus grayscale for AI may not mirror human diagnostic priorities. Historically, many histological structures and organisms were recognizable in monochrome atlases, supporting the idea that shape, spacing, and clustering can be highly informative even without hue. Accordingly, future work should explore routine training using both grayscale and color-only representations, which can be generated efficiently via YCbCr (or LAB), to assess complementary contributions of luminance and chromatic information.

Looking forward, the generalizability of these findings likely depends on the diagnostic task. Nuclear- or morphology-centric tasks (e.g., pleomorphism grading, mitotic detection, tumor cellularity, and tumor–stroma ratio) are expected to behave similarly, as they rely predominantly on luminance-encoded structure. In contrast, tasks that depend more strongly on chromatic cues, such as evaluation of necrosis, inflammation, hemorrhage, microorganisms (e.g., acid-fast bacilli or fungi), or certain cytoplasmic secretory or extracellular components, are more likely to be color-sensitive. Accordingly, these results do not imply that color is universally dispensable in histopathology; rather, they suggest that for structurally driven, H&E-based tasks, luminance tends to dominate both human perception and AI performance. These conclusions, therefore, apply specifically to H&E-stained images with a limited color palette and may not extend to stains in which color conveys stronger diagnostic meaning, such as immunohistochemistry, special stains, or fluorescence-based staining.

In addition, no formal human-observer validation was performed. Although such studies would complement the present findings, this work focused on an objective, large-scale AI-based assessment. Whereas Campbell et al.^46^ demonstrated the influence of color on human diagnostic interpretation in surgical pathology, systematic investigation in this area remains limited. Finally, although XCC showed minimal perceptual and functional impact in this study, its implementation in routine digital pathology workflows will require further validation within WSI, telepathology, and regulatory frameworks.

## Conclusion

Within the constraints of this study, and using AI as an objective, scalable surrogate observer, our findings indicate that, for a morphology-driven, clinically central task in H&E-stained material, diagnostic performance is largely preserved when luminance information is retained, and chromatic information is altered or substantially reduced. Color remains essential for visual comfort and efficient human interpretation; however, in this context, it appears to contribute less to the core diagnostic signal than structural information encoded by luminance. These observations suggest that, for selected tasks and stains, color manipulation or compression may be feasible with minimal impact on diagnostic integrity, and that color normalization may warrant contextual rather than universal emphasis. Nevertheless, these conclusions are specific to the experimental framework used and should not be extrapolated to all diagnostic tasks, stains, or clinical settings. Further studies, including broader histopathological evaluation, are required to define the general applicability of these findings.

## Declaration of generative AI and AI-assisted technologies in the manuscript preparation process

During the preparation of this work, the author(s) used ChatGPT in order to proofread the manuscript. After using this tool/service, the author(s) reviewed and edited the content as needed and take(s) full responsibility for the content of the published article.

## Patient consent and ethical approval

Not applicable.

## CRediT authorship contribution statement

ER and LD prepared the manuscript and approved the final version. IOE approved the manuscript.

## Funding

No fund was available.

## Declaration of competing interest

The authors declare that they have no known competing financial interests or personal relationships that could have appeared to influence the work reported in this article.

## Appendix A Supplementary data

Supplementary material 1 Supplementary Material S1. Extended methodological description of colour manipulation, extreme colour compression (XCC), colour-blind-friendly image generation, deep learning modelling, and statistical analyses. Supplementary material 1

Supplementary material 2 Supplementary Table S1. Cross-table of p-values for pairwise comparisons of ΔAUC between different colour image representations. Supplementary material 2
