# Supplementary material for: Structure over color: Diagnostic information in H&E images resides primarily in grayscale
Source: J Pathol Inform. 2026 Feb 5;21:100646. doi: 10.1016/j.jpi.2026.100646 (PMC12991843; doi:10.1016/j.jpi.2026.100646)
Supplement: Supplementary material 1 — Supplementary Material S1. Extended methodological description of colour manipulation, extreme colour compression (XCC), colour-blind-friendly image generation, deep learning modelling, and statistical analyses. [file mmc1.docx]

**METHODS**

Colour alterations were performed by converting RGB images into the YCbCr colour space ^1,2^. YCbCr has been used for several decades and has a well-established historical background. It was developed during the transition from black-and-white to colour television, at a time when colour receivers were not universally available. In this system, legacy black-and-white televisions processed only the luminance (Y) signal, whereas colour televisions additionally decoded the chrominance components (Cb and Cr). This separation of luminance and chrominance makes YCbCr particularly well suited for independent manipulation of structural (grayscale) and colour information.

Figure 1 illustrates a representative breast cancer image and the corresponding intensity histograms for the three YCbCr components. Notably, the chrominance channels (Cb and Cr) occupy a relatively narrow range of intensity values, with most of the available 256 levels unused, whereas the luminance (Y) channel spans a broad dynamic range. This property enabled the generation of colour-blind-friendly (CBF) images by redistributing red–green information into unused intensity levels within the Cb channel.

Figure 2 shows the same image decomposed into its Y, Cb, and Cr components. The Y channel appears as a conventional grayscale image, while the Cb and Cr channels, when viewed independently, also appear as grayscale images. Although stored as standard 8-bit grayscale images, the Cb and Cr values are interpreted upon recombination with Y as chromatic shifts along the blue–yellow and red–green axes, respectively. The Y channel defines image brightness, while Cb and Cr modulate hue.

When the Y channel is fixed at a single intensity value, the image contains no structural contrast. Recombining this constant Y with the original Cb and Cr channels yields what we refer to as a colour-only image, which represents the conceptual inverse of grayscale. An example of a colour-only image is shown in the lower right panel of Figure 2.

**Colour-blind-friendly image:**

There is no universally accepted standard for defining a colour-blind-friendly (CBF) palette for H&E images. Most established CBF approaches have been developed for graphs and data visualisation rather than histopathology ^3,4^. Grayscale images have traditionally been used for teaching histology to colour-blind students ^5^, and a CBF approach specifically tailored to pathology has also been previously described ^6^. The method presented here differs in its explicit use of the YCbCr colour space to redistribute chromatic information.

Figure 3 illustrates an example of a CBF image generated using this approach. Differential information from the Cr (red-green) channel was transferred into unused intensity ranges of the Cb channel, after which Cr values were set to a constant. As a result, the Cr channel becomes effectively uniform, appearing as a “blank sheet of grey paper” in the lower-left panel. In contrast, the Cb channel shows marked information redistribution compared with the original Cb image in Figure 1, with nuclear and architectural features remaining readily discernible.

**Extreme colour compression:**

It is well established that excessive image compression can degrade image quality [Gonzalez]. If colour carries essential diagnostic information in H&E images, then aggressive compression of colour channels would be expected to affect image quality and interpretability adversely. The YCbCr colour space enables independent compression of luminance (Y) and chrominance (Cb, Cr), allowing colour information to be compressed separately from grayscale structure. Accordingly, the Cb and Cr components can be compressed at one compression ratio (cratio), while the Y component is compressed at another.

In Figure 4, the Y channel was left uncompressed. In contrast, the Cb and Cr channels were compressed at a 1000:1 ratio. We refer to this approach as eXtreme colour compression (XCC), and the level of compression applied is far beyond that used in standard image formats. By comparison, conventional JPEG compression typically averages four colour pixels per luminance pixel (chroma subsampling) and likewise relies on the YCbCr colour space ^7^. The widespread use of YCbCr underscores its robustness and suitability for colour manipulation.

For this study, compression was performed using JPEG2000 ^7^, which permits precise control of compression ratios and independent handling of individual channels, thereby avoiding the block artefacts characteristic of standard JPEG compression. JPEG2000-based compression has been previously evaluated in the context of digital pathology ^8^.

Figure 4 illustrates the effects of extreme compression applied to grayscale and colour-only images. When grayscale images were compressed aggressively, image quality was visibly degraded, with cytological detail appearing “smudged.” In contrast, the lower-left panel of Figure 4 shows an image with extreme compression applied only to the colour channels, while the grayscale channel remained uncompressed. Compared with the original image (Figure 1), no perceptible loss of image quality was observed.

To explore the limits of acceptable visual quality, XCC was further extended using a Y cratio of 60 and a Cb/Cr cratio of 2000. At Y = 60, the multi-scale structural similarity index (MS-SSIM) relative to the original image was 0.947. A dataset of 100 lossless PNG images occupied 518 MB; after XCC processing, the compressed Cb and Cr components were 390 KB each, and the Y component was 13.3 MB, corresponding to 2.7% of the original storage size. Notably, colour information accounted for only 0.15% of the total compressed data volume, demonstrating that colour can be compressed extremely efficiently with minimal impact on image quality.

**Comparison of all.**

Figure 5 presents side-by-side comparisons of all image alterations, including eXtreme colour compression (XCC), grayscale, colour-blind-friendly (CBF), and colour-only images. Additional examples, including images from cancers arising in different organs, are provided in the Supplementary Material. Further examples are available from the corresponding author (LD) upon reasonable request.

**Python libraries.**

All image processing was performed using Python (version 3.10). The Pillow (PIL) library ^9^ was used for image input and output, RGB-to-YCbCr colour-space conversion, splitting and recombining individual channels, and generating grayscale representations of the Y, Cb, and Cr components. JPEG2000 compression was implemented using the glymur library ^10^.

**Image quality metrics.**

In many applications, it is impractical for human observers to assess the quality of all generated images visually. To address this limitation, a range of image quality metrics (IQM) algorithms have been developed to approximate human perceptual assessment ^11^. These algorithms are typically calibrated using human observer data, enabling automated evaluation once trained. Numerous IQM methods have been proposed, and the selection of an optimal metric remains an area of ongoing debate ^12^. In this study, image quality was quantified using the multi-scale structural similarity index (MS-SSIM) ^13,14^, implemented via the sewar Python library (version 0.4.6).

**Deep Learning (AI) models**

The various colour alterations were also evaluated for their functional impact by comparing the performance of deep learning models trained and tested on each altered image set with that of models trained on the original full-colour images. All convolutional neural network models were trained using procedures consistent with prior work ^15^, with the pretrained architecture updated to ConvNeXt ^16^.

Whole-slide images (WSIs) were obtained from The Cancer Genome Atlas (TCGA) via the Cancer Digital Slide Archive ^17^. The training set comprised 192 breast cancer cases contributed by the University of Pittsburgh, Walter Reed National Military Medical Centre, and Roswell Park Comprehensive Cancer Centre. From these training WSIs, 105,881 image tiles (250 × 250 pixels) were randomly extracted, of which 3,516 high-grade, 3,312 low-grade, and 3,128 benign tiles were selected by pathologists.

The test set consisted of slides from all remaining institutions (N = 271), yielding a total of 95,952 image tiles. There was no institutional overlap between the training and test datasets. As is well recognised for TCGA material, substantial inter-institutional variability in staining was present.

The modelling task was to estimate the probability that an image tile contained high-grade nuclei, particularly those demonstrating nucleolar prominence ^18^. This is illustrated by representative heatmaps in Figure 6. Tiles classified as positive for high-grade nuclei were counted, and the fraction of positive tiles (frachigh) was used as the slide-level predictor.

A ConvNeXt-Tiny network (TensorFlow/Keras implementation pretrained on ImageNet) was employed for three-class classification. Images were resized to 224 × 224 pixels, converted to RGB format, and rescaled to the 0–1 range. An 80/20 training–validation split was applied (random seed = 42). Data augmentation included rotations, translations, shear, zoom, and horizontal and vertical flips. The ConvNeXt backbone was frozen, and a classification head consisting of global average pooling, a dense layer with 128 units (ReLU activation) and dropout (0.5 and 0.3), and a three-unit softmax output layer was trained. Models were optimised using the Adam optimiser (learning rate 1 × 10⁻⁴), sparse categorical cross-entropy loss, batch size of 8, and a maximum of 9 epochs, with early stopping and learning-rate reduction applied.

Statistical analyses were performed in R ^19^. Spearman rank correlations were calculated using base R, with bootstrap confidence intervals generated using the boot package ^20,21^. Receiver operating characteristic (ROC) curves were constructed using the pROC package ^22^. The response variable was pleomorphism score 3 (high nuclear grade), and predictors were the model-derived probabilities of high-grade classification for each colour alteration. Differences in AUCs, where present, were assessed by bootstrap resampling. Figures were generated using ggplot2 ^23^.

**Normalisation of brightness.**

Given that colour primarily serves an aesthetic role, normalisation can be confined to the grayscale (luminance) component alone. The advantage of this approach is that adjustment is restricted to brightness, avoiding direct manipulation of chromatic information. A simple and effective method is to compute the median grayscale value across all images and scale each image to this common median. The original chrominance components (Cb and Cr) are left unchanged. Any perceived change in colour, therefore, arises indirectly from changes in underlying brightness rather than from explicit colour modification.

Using this approach, the multi-scale structural similarity index (MS-SSIM) between the original and brightness-normalised images (without compression) remained essentially unchanged (MS-SSIM = 0.99), indicating negligible alteration of structural content. Figure 7 illustrates an example of increased image brightness following grayscale normalisation.

Accordingly, grayscale information was used both for image normalisation and for defining compression levels in this study.

**References**

1. Ramanath R, Drew MS. Colour spaces. In: Computer Vision. Cham: Springer; 2020. doi:10.1007/978-3-030-03243-2_452-1.

2. Gonzalez RC, Woods RE. Digital Image Processing. 4th ed. New York: Pearson; 2018.

3. Wong B: Color blindness. Nat Methods 8:441, 2011

4. Okabe M, Ito K. Colour universal design (CUD): how to make figures and presentations that are friendly to colourblind people. Tokyo: University of Tokyo; 2002.

5. Rubin LR, Lackey WL, Kennedy FA, et al: Using color and grayscale images to teach histology to color-deficient medical students. Anat Sci Educ 2:84-8, 2009

6. Landini G, Perryer G: Digital enhancement of haematoxylin- and eosin-stained histological images for red-green colour-blind observers. J Microsc 234:293-301, 2009

7. Wallace GK. The JPEG still picture compression standard. Communications of the ACM, Volume 34, Issue 4. Pages 30 - 44. <https://doi.org/10.1145/103085.103089>.

8. Ghazvinian Zanjani F, Zinger S, Piepers B, et al: Impact of JPEG 2000 compression on deep convolutional neural networks for metastatic cancer detection in histopathological images. J Med Imaging (Bellingham) 6:027501, 2019

9. Clark A. Pillow: The Python Imaging Library (PIL Fork) Documentation. 2023. Available from: <https://python-pillow.org/>.

10. Evans J. Glymur: a Python interface for JPEG 2000 [software]. Version 0.14.4. 2025. Availa-ble from: <https://github.com/quintusdias/glymur>.

11. Wang, Zhou and Alan Conrad Bovik. “Modern Image Quality Assessment.” Modern Image Quality Assessment (2006). <https://api.semanticscholar.org/CorpusID:42379352>.

12. Renieblas GP, Nogues AT, Gonzalez AM, et al: Structural similarity index family for image quality assessment in radiological images. J Med Imaging (Bellingham) 4:035501, 2017

13. Afnan, Ullah F, Yaseen, et al: Subjective Assessment of Objective Image Quality Metrics Range Guaranteeing Visually Lossless Compression. Sensors (Basel) 23, 2023

14. Sheikh HR, Sabir MF, Bovik AC: A statistical evaluation of recent full reference image quality assessment algorithms. IEEE Trans Image Process 15:3440-51, 2006

15. Elsharawy KA, Gerds TA, Rakha EA, et al: Artificial intelligence grading of breast cancer: a promising method to refine prognostic classification for management precision. Histopathology 79:187-199, 2021

16. Liu Z, Mao H, Wu CY, Feichtenhofer C, Darrell T, Xie S. A ConvNet for the 2020s. In: Proc IEEE/CVF Conf Comput Vis Pattern Recognit. 2022:11966–11976.

17. Gutman DA, Cobb J, Somanna D, et al: Cancer Digital Slide Archive: an informatics resource to support integrated in silico analysis of TCGA pathology data. J Am Med Inform Assoc 20:1091-8, 2013

18. Elsharawy KA, Toss MS, Raafat S, et al: Prognostic significance of nucleolar assessment in invasive breast cancer. Histopathology 76:671-684, 2020

19. R Core Team. R: A Language and Environment for Statistical Computing. R Foundation for Statistical Computing, Vienna. 2021. <https://www.R-project.org>.

20. Canty A, Ripley BD. boot: Bootstrap R (S-Plus) Functions. R package version 1.3-30. 2024.

21. Davison AC, Hinkley DV. Bootstrap Methods and Their Applications. Cambridge: Cambridge University Press; 1997.

22. Robin X, Turck N, Hainard A, et al: pROC: an open-source package for R and S+ to analyze and compare ROC curves. BMC Bioinformatics 12:77, 2011

23. Wickham H. ggplot2: Elegant Graphics for Data Analysis. New York: Springer; 2016.
