# Supplementary material for: Structure over color: Diagnostic information in H&E images resides primarily in grayscale
Source: J Pathol Inform. 2026 Feb 5;21:100646. doi: 10.1016/j.jpi.2026.100646 (PMC12991843; doi:10.1016/j.jpi.2026.100646)
Supplement: Supplementary material 2 — Supplementary Table S1. Cross-table of p-values for pairwise comparisons of ΔAUC between different colour image representations. [file mmc2.docx]

**Supplementary Table 1.** Cross‐table of p-values corresponding to ΔAUC for the different colour types.

|  | **Original** | **CO** | **Grayscale** | **CBF** | **XCC** |
| --- | --- | --- | --- | --- | --- |
| **Original** |  | 0.052 | 0.29 | 0.02 | 0.77 |
| **CO** | 0.052 | --- | 0.027 | 0.0008 | 0.075 |
| **Grayscale** | 0.29 | 0.027 | --- | 0.16 | 0.34 |
| **CBF** | 0.02 | 0.0008 | 0.16 | --- | 0.05 |
| **XCC** | 0.77 | 0.075 | 0.34 | 0.05 | --- |

CO = colour only; CBF = colour-blind friendly; XCC = extreme colour compression.

There was a highly significant difference in ΔAUC between CO and CBF for reasons that are not currently understood. CO showed borderline differences with several other colour variations. Among Original, Grayscale, and XCC, no significant differences were observed. CBF showed no significant difference compared with Grayscale and demonstrated borderline significance when compared with Original and XCC.
